# Developing A General Framework for National Health Information Network for Developing Countries

**DOI:** 10.31661/gmj.v9i0.1792

**Published:** 2020-12-29

**Authors:** Hamid Moghaddasi, Reza Rabiei, Farkhondeh Asadi, Ali Mohammadpour

**Affiliations:** ^1^Health Information Management and Medical Informatics, Health Information Technology and Management Department, Faculty of Paramedical Sciences, Shahid Beheshti University of Medical Sciences, Tehran, Iran; ^2^Medical Informatics, Health Information Technology and Management Department, Faculty of Paramedical Sciences, Shahid Beheshti University of Medical Sciences, Tehran, Iran; ^3^Health Information Technology and Management Department, Faculty of Paramedical Sciences, Shahid Beheshti University of Medical Sciences, Tehran, Iran; ^4^Department of Health Information Technology, School of Paramedical Sciences, Hamadan University of Medical Sciences, Hamadan, Iran

**Keywords:** National Health Information Network, Health Information Exchange, Health Information Systems

## Abstract

**Background:**

The National Health Information Network (NHIN) is one of the key issues in health information systems in any country. However, the development of this network should be based on an appropriate framework. Unfortunately, the conducted projects of health information systems in the Ministry of Health of Iran do not fully comply with the concept of NHIN. The present study was aimed to develop a general framework for NHIN in Iran.

**Materials and Methods:**

In this study, in the first stage, the required information about the concept of the NHIN framework and related NHIN documents in the USA and the UK were collected based on a literature review. Then, according to the results of the first stage and with regards to the structure of the Iranian health system, a general framework for Iranian NHIN was proposed. The Delphi technique was conducted to verify the framework.

**Results:**

The proposed framework for Iranian NHIN includes three dimensions; components, principles, and architecture. Over 80% of experts have evaluated all three aspects of the framework at an acceptable scale. In total, the proposed framework has been evaluated by 83.8% of the experts at an acceptable scale.

**Conclusion:**

The proposed framework was expected to serve as the starting point for moving towards the design and creation of Iranian NHIN. At any rate, the framework could be criticized, and it could only be used for the countries whose health system is similar to the structure of the health system in Iran.

## Introduction

Nowadays, even though the Ministry of Health (MoH) is the protector of public health and responsible for collecting, processing and using health data nation-wide, there are some other departments involved in the health domain and information [1-3]. There is a significant relationship between the health information systems of MoH and information systems of other departments and organizations, including Atomic Energy Organization, Ministry of Interior, Ministry of Defense, Ministry of Industry, and Ministry of Agriculture [3-4]. Considering these relationships, the National Health Information Network (NHIN) notion has emerged [5-6]. NHIN is a network that relates all health service providers, health plans and projects, health information providers, governmental organizations, and health-related organizations to exchange health information [6]. NHIN is expected to collect health data from different systems and share them among the stakeholders. Using these data, the health activists, especially from MoH, can monitor the whole nation’s health problems meticulously [1,6-7]. Developed countries have highlighted the importance of NHIN for many years. To develop NHIN, the US and the UK have taken significant steps since the early 2000s. Their projects were entitled NHIN and National Programme for Information Technology (NPfIT) in the National Health Service(NHS) [6,8-9]; whereas, studies in developing countries show that lack of policies and comprehensive plans on national health information is a serious deficiency in these countries [10-11]. In Iran, review of the documents related to projects on health information systems of MoH including Electronic Health Record (SEPAS), National Health Network (SHAMS) and Integrated System of Iran’s Health Statistics and Information System (SINASA) [12-15], and their assessment based on NHIN’s definitions in valid texts [6] demonstrate that the above-mentioned projects do not have full compliance with the concept of NHIN. Generally, in these projects, an intra-organizational perspective is a priority because SEPAS acts as the connector between hospital information systems and SHAMS is considered as the communication infrastructure of medical and diagnostic centers [13-14]. In contrast, SINASA is an integrated database of statistics and information of MoH [15]. Review of National Scientific Health Plan (2007), Health System Reform Plan (2011) and other documents, including the Fifth National Development Plan in the health domain, show that NHIN in Iran is not taken seriously as it should be [16-18]. National studies also indicate some deficiencies of the National Health Information System (NHIS), including lack of standards, laws, regulations, and inappropriate infrastructure [3]. Besides, in the evaluation of the Iranian National Health System, the world bank pointed out the strengths and weaknesses of Iranian health information systems and suggested some advice to improve the NHIS. The need for designing NHIN was notable [19]. In general, insufficient attention to developing NHIN in Iran is a remarkable deficiency [12-16]. On the other hand, it is evident that to achieve the ultimate goal of health improvement and other advantages of developing NHIN; it is essential to design it based on an appropriate framework [4,20]. In this framework, Stead *et al.* (2005) mentioned some issues such as governance, policy, and architecture of the network [4]; moreover, the Office of National Coordinator for Health Information Technology (ONC) in the US emphasized on the architecture of NHIN [21-22]. Moreover, the Health Metrics Network (HMN) and its stakeholders suggested a framework for developing the NHIS. From an HMN standpoint, the NHIS framework entails components, principles, and processes that form NHIS [20]. However, the health system structure is unique for each country, and a common framework cannot be proposed to all countries. On the other hand, unfortunately, the conducted projects of health information systems in the MoH of Iran do not have full compliance with the concept of NHIN, and it is a remarkable deficiency in the field of health information systems of Iran. Therefore, the present study aimed to develop a general framework for the NHIN of Iran.

## Materials and Methods

In the first phase of this applied study, based on the literature review, the concept of the NHIN framework was defined in three dimensions; components, principles, and architecture. Secondly, the documents related to the NHIN project of the US and the UK were obtained through the review of the scientific databases as well as the MoH website of these countries. Then, the documents were analyzed regarding the dimensions in the NHIN framework. In the second phase of the study, using the information of NHIN framework of the afore-mentioned countries, valid scientific literature and considering the structure of Iran’s health system [23-25], a framework was suggested for Iranian NHIN, and then the Delphi method [26] was conducted to verify the framework. In conducting the technique, we used some experts, including ten specialists in the field of medical informatics and health information management. They were all faculty members of Iranian universities holding the rank of assistant professor and higher, and they had more than ten years of job experience. Besides, some other experts were previous and present officials of the Information Technology and Statistics Office of Iranian MoH. In the second phase of the study, a semi-structured questionnaire was used for data gathering, and its validity was assessed through the specification of content validity and the reliability was interpreted by Cronbach’s Alpha method (α=0.88). The questionnaire was divided into three sections, including framework components, principles, and architecture, with nineteen, five and three close-ended questions, respectively. Furthermore, at the end of each closed-ended question, there was an open-ended question for the experts’ comments. Each closed-ended question was designed with multiple choices of “acceptable,” “relatively acceptable,” and “unacceptable,” with the numerical value of 3, 2 and 1, respectively. Thus, regarding ten participants in the Delphi technique, each question’s maximum and minimum scores could be 30 and 10, respectively. Hence, according to these maximum and minimum scores, the acceptance, rejection, and revision of each item were as follows: each item score ≥25 = acceptance; 20 ≤ each item score ˂ 25 = revision; each item score ˂ 20 = rejection. Distributing and collecting the questionnaires were done both in person and through e-mails. Finally, data were analyzed using SPSS-13 software.

## Results

 The results of the study are presented in two sections: 1) review and comparison of the NHIN framework in the afore-mentioned countries and proposal for Iranian NHIN; 2) verifying and developing a general framework for Iranian NHIN.

###  Review and Comparison of NHIN Framework of the US and the UK, and Suggestion for Iranian NHIN

Tables-1 and 2 respectively depict the comparison of the principles and components of the NHIN framework in the afore-mentioned countries. The architecture of NHIN in these countries and the suggested architecture for Iranian NHIN are addressed. In this study, the NHIN architecture includes three dimensions of network members, interfaces (interactions between members), and communication with the environment (security and confidentiality) [21,27-28]. According to this definition, the assessment of NHIN architectures showed that both countries are almost similar in developing interfaces and adjusting the security and confidentiality of NHIN. They both defined the gateways for the connections of members [21,29-30] and communication with the environment was set by autonomy and local responsibility, technical strategies, and setting up the legal agreement for exchanging and using health data [31-33]. Besides, regarding the members of the network, it can be said that NHIN in the US is formed by the connection of Health Information Exchange centers, Regional Health Information Organizations, integrated delivery networks, state-wide health information exchange programs, federal agencies (including 33 agencies and national organizations), state and local governments, hospitals, clinics, drug stores, laboratories, imaging centers, insurance, and reimbursement system, local health centers and health care organizations [21-22]. In the UK, members of the N3 include Community of Interest Networks (CoINs), gateways to other networks (including gateways of internet, phone and mobile network, pharmaceutical network, academic network, gateways to the National Health System in Wales and Northern Ireland, the gateway to government network including government departments and local authorities and agencies) and direct members (including acute ambulance and care trusts, dentists, foundation trusts, general practitioners, health system and software providers, hospice centers, independent health care sector, local authorities, mental health trusts, national blood service, systems of Health and Social Care Information Center [HSCIC], insurance and reimbursement systems, primary care trusts, special health authorities) [30,32]. The suggested architecture for Iranian NHIN will be explained below.

###  Developing and Verifying a General Framework for Iranian NHIN

**Figure 1:**
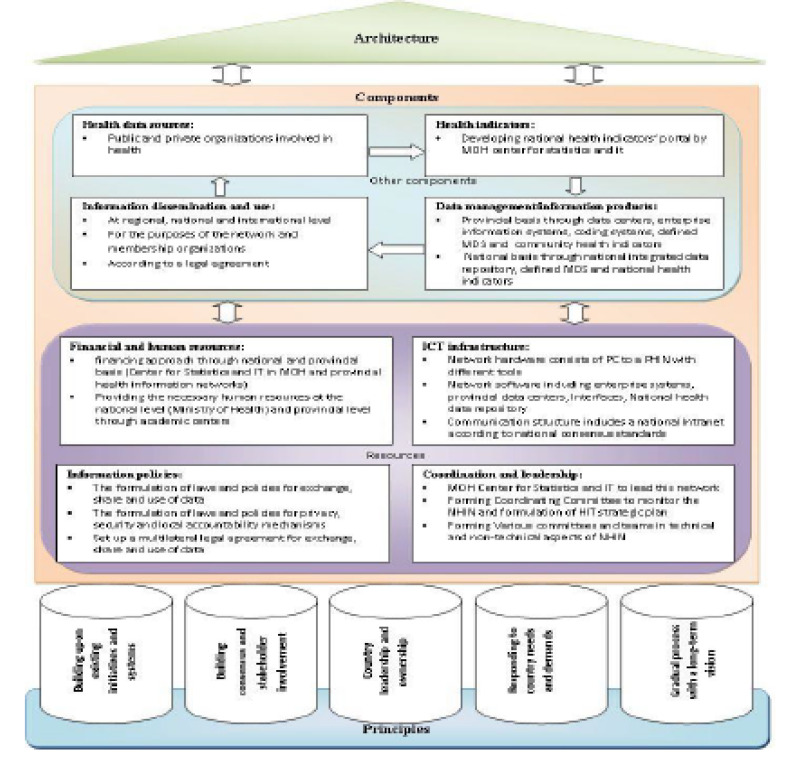
A suggested framework for Iranian NHIN

**Figure 2:**
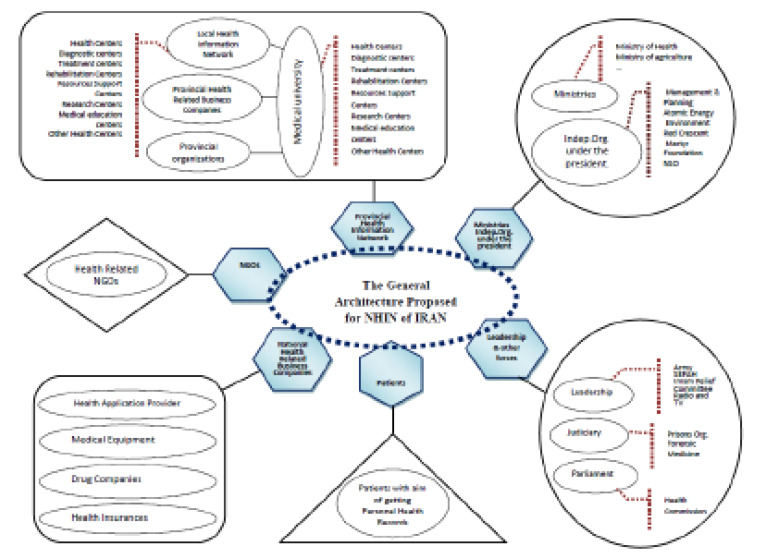
A suggested architecture for Iranian NHIN

 The findings related to the suggested framework and the architecture of Iranian NHIN are depicted in Figures-1 and 2, and experts’ opinions on this framework are demonstrated in Tables-3, 4, and 5. According to Figure-1, the suggested framework for Iranian NHIN has three dimensions; components, principles, and architecture. The components include eight key elements. The principles consist of five essential principles that work as the infrastructure of two other dimensions (the components and the architecture). The architecture comprises of network members/nodes, interfaces, and communication with the environment. According to Figure-2, the foundation of the suggested architecture for Iranian NHIN entails provincial health information networks. The interfaces involve gateways that will be developed by private and public sector organizations based on their needs, standards and technical requirements. Communication of NHIN with the environment will be set based on technical requirements, multilateral agreement of exchanging, sharing and using health data and information, and local authority. Table-3 and 4 show the frequency distribution of experts’ opinions and scores of items in each dimension of the proposed framework. Overall, according to the findings of Table-5, the proposed framework was assessed by 83.85, 15.95, and 0.2 percent of the experts on an acceptable, relatively acceptable, and unacceptable scale, respectively.

## Discussion

The main objective of the present study was to develop a general framework for the NHIN of Iran. To achieve this goal, we tried to consider valid scientific literature, the framework developed by the health metrics network [20] and the approaches related to NHIN architecture [34-35]. Also, the experiences of the two leading countries in the development of NHIN, namely, the US and the UK, and the structure of Iran’s health system were considered. Based on the analysis of the findings related to the comparison of the NHIN framework in the studied countries, it can be said that generally, developing NHIN in the US is adjusted with the decentralized approach while it is adjusted with the centralized approach in the UK [9,21]. In the NPfIT project in the UK, sovereignty, launching the systems, and specifying the members of the N3 network were entirely defined as centralized [9,32]. However, in the NHIN project of the US, most of the issues in dimensions of the components, the architecture, and the principles of the NHIN framework were decentralized [21,36]. The weaknesses of the centralized approach of the NPfIT program are insufficient attention to local needs and end-users, and lack of flexibility versus technological changes. However, high data quality, better data use and management, and ultimately better control on the budget spending are the strengths of this approach [8,34-35]. In the approach used by the US in the NHIN program, low data quality and minimal use of data at the national level are among the weaknesses. There are also strengths in this approach, including focusing on local needs and end-users, and flexibility versus technological changes [34-35]. In any case, the development of Iranian NHIN has been done to minimize the weaknesses in the two approaches mentioned. To achieve this goal, many items related to the components and the architecture dimensions in the suggested framework are proposed as a combination of national and provincial levels. On the other hand, the Middle Out approach [34] has also been at the forefront. Given that the main basis of the geographical divisions of the country is the province, and on the other hand, the health responsibility of each province lies with the medical universities, and at the national level, this role is played by the MoH [23-25]. Therefore, components such as financial resources, software applications, and data management are proposed as a combination of national and provincial levels to address local and national needs and exist some kind of competition among PHINs. In some cases, including the communication structure in the information and communications technology (ICT) resource element, due to the state of the country’s communications infrastructure, including Internet service problems, the National Intranet has been proposed to be in the best position to respond to the network in the long run. This is somewhat similar to the situation in the UK. The study’s findings conducted by Damanabi et al. (2014) are also consistent with the proposed components in this framework. She grouped the inputs of the Iranian NHIS into eight dimensions; data sources, coordination and leadership, information policies, human resources, financial resources, facilities, information and communication infrastructure, and, finally, the cultural and institutional aspects [37]. In general, based on the literature review, there are three approaches to NHIN architecture [34-35]. The NHIN architecture in the US, which involves connecting regional health information networks and other health-related organizations and centers, is known as the bottom-up approach [34-35]. This approach is unlike the type of architecture used on the N3 network from the UK’s NPfIT program. The N3 network members and its information systems are centrally defined, which is known as a top-down approach [8,34-35]. Conducted studies, in addition to these approaches, have also referred to a third approach called “Middle -Out” [34]. In this approach, the needs of healthcare providers, the IT industry and government are considered, and then common goals are defined for technical and non-technical issues of NHIN. The government takes the lead in developing the network and plays a facilitating role. Then, by defining the interoperability standards, the NHIN is formed by connecting PHINs and other stakeholders [34]. Based on the findings of Coiera et al. (2009), given the large scale of the NPfIT project, the NHS top-down approach cannot adapt quickly to the challenges associated with providing health services. In contrast, the bottom-up approach is robust against severe changes, including new technologies or re-engineering of systems [34]. Note that in the NHIN projects of the countries under review, some changes have been made to the Middle-Out approach. In the US, due to the inability to finance RHIOs and the lack of attention to the needs of some states, the State Health Information Exchange Programs was eventually introduced in 2010 [36], and in the UK, in the middle of the NPfIT program, the CoINs and local ownership was also introduced [9,29]. However, the selection of any of these approaches depends on the structure and the nature of the health system of a country. The health system is relatively centralized in some countries, and in other countries, it is completely decentralized. Indeed, the type of NHIN architecture in these types of systems will be somewhat different [34]. Given the above-mentioned points and the fact that the main structural blocks of the Iranian health system are medical universities in each province, and this structure is more compatible with the Middle-out approach, this approach was used in the NHIN architecture of Iran. The main foundation of this architecture is provincial health information networks led by each province’s medical universities. The findings of Targowski *et al*. (2011) study are consistent with the findings of the proposed architecture of the present study. He has developed the NHIN architecture in the US at four levels, including local, regional, national and international levels. The local level refers to service providers at the local level; the regional level includes state governments, health centers, and regional organizations. The federal government, federal agencies, centers, and national organizations are also at the third level nationally. The fourth level also refers to service providers who provide health services to citizens of other countries [38]. The principles of the proposed framework are presented within the framework of the HMN and were approved by more than 100 ministers and responsible officials of international agencies [20]; therefore, suggested as the guiding principles for developing the NHIN of Iran, and approved by experts. According to the findings of Table-4, 80% of experts have evaluated these principles at an acceptable scale, indicating acceptance of these principles at a high level. Overall, the conclusion and analysis of expert’s opinions about the acceptability of each aspect of the proposed framework, and finally, the acceptable rate of the proposed framework (83.85%) suggests confirmation of the proposed framework by an overwhelming majority of experts and demonstrates the strength of the proposed framework. It is expected that the proposed framework will lead to greater coordination among stakeholders of information and health services across the country; besides, it serves as the basis for all projects of the Ministry of Health to manage the health information of the country. In other words, the framework can serve as a starting point to move towards the design and creation of NHIN in Iran. In this regard, suggestions are made for the development of NHIN in Iran as follows:

 - All ministries and stakeholders of the health realm must confirm the IT and Statistic Center of MoH as the leader of all activities related to national health information and statistics.

 - Compiling the national plan of health IT to design and develop NHIN with the collaboration of all stakeholders.

 - Specifying the role and responsibilities of stakeholders of the network and providing instruction on how to use data and information according to the legal agreement.

 - Specifying significant resources for developing NHIN.

 - Specifying the members as producers and users of health data considering the suggested architecture.

 - Specifying how to manage the data,

 - Assessing the suggested architecture and modifying SEPAS and SHAMS projects based on it; besides, it is needed to consider the proposed principles as the red lines in designing and developing the network. On the other hand, given the steps in developing this framework, especially considering different NHIN design approaches, countries with government and health system structures like Iran, in developing NHIN can use this framework as a road map.

## Conclusion

 The development of NHIN does not just mean setting up national systems or systems or connecting local systems, but also it is needed to have a framework in which most issues related to NHIN formation are addressed. The experiences of two leading countries in developing NHIN, approaches in designing NHIN, and the structure of the Iranian health system was considered in the suggested framework. The framework is expected to serve as the starting point to move towards the design and creation of Iranian NHIN. At any rate, the suggested framework could be criticized, and it could only be used for the countries having a structure similar to the Iranian health system.

## Acknowledgment

 We would like to thank Dr. Sinaei and Mr. Omid Baan, EFL/ESL Instructor, Farsi-English & English-Farsi Translator/Interpreter, for their assistance in writing this paper.

## Conflict of Interest

 The authors declared no potential conflicts of interest concerning the research, authorship, and/or publication of this article.

**Table 1:** Comparison of the Principles Dimension of NHIN Framework in the US and the UK and Suggestion for Iranian NHIN

**Country** **Principles**	**United States** **(NHIN)**	**United Kingdom** **(NPfIT)**	**Iran** **(NHIN)**
**Country leadership and ownership**	ONC(office of the national coordinator for HIT)	HSCIC(health and social care information center)	Center for IT and Statistics
**Responding to country needs and demands**	NHIN Coordination Committee + compiling a national strategic plan for HIT + Published RFI, RFP	Partially focused on needs + compiling strategy for HSCIC	NHIN Coordination Committee + national strategic plan for HIT+ Various committees and teams
**Building upon existing initiatives and systems**	NHIN as a network of networks, without replacing other systems	Because of the centralized view in NPfIT, this principle has not been observed.	NHIN as a network of networks
**Building consensus and stakeholder involvement**	NHIN Coordination Committee	In NPfIT this principle is not observed.	NHIN Coordination Committee
**Gradual process with a long-term vision**	Compiling national strategic plan for HIT	Substantial changes during the program	Compiling national strategic plan for HIT

**Table 2:** Comparison of the Dimension of the Components of the NHIN Framework in the US and the UK and Suggestion for Iranian NHIN

**Country** **Components**	** United States** **( NHIN ) **	** United Kingdom** **( NPfIT ) **	**Iran** **(NHIN)**
**Leadership and coordination**	ONC + Coordination Committee + federal advisory committees + HIT strategic plan	HSCIC + managerial Board of the Ministry of Health + strategy of HSCIC	Center for IT and Statistics + Coordinating Committee+ Various committees and teams+ HIT strategic plan
**Information policies**	DURSA + federal and state laws related to Information security and privacy	Information Governance Statement of Compliance (IGSoC)	Formulation of laws and policies for data use, privacy, security, and local accountability Legal agreement
**Financial resources**	Decentralized	Centralized	National and provincial basis
**Human resources**	Training human resources by universities + certified exam by ONC	Training human resources by HSCIC and universities	Human resources at the national and provincial levels through academic centers
**ICT**	**Hardware**	Computers+ network equipment	Computers + network equipment	Computers + network equipment
	**Software**	NHIN gateway+ federal information systems + member’s information systems	N3 gateways + national systems	Enterprise systems, provincial data centers, interfaces, national health data repository
	**Infrastructure**	Internet	private WAN + Internet	National Intranet
**Health Indicators**	National Health Indicators	National Health Indicators Portal	National Health Indicators’ Portal
**Health data sources**	As members of NHIN	As members of N3	Public and private organizations involved in health
**Data management**	Locally + National Health indicators Portal + definition of minimum data set (MDS)	Nationally + National Health indicators Portal + definition of MDS	Provincial basis: data centers, coding systems, defined MDS and community health indicators National basis: national integrated data repository, defined MDS and national health indicators
**Information dissemination** **and use**	By DURSA agreement, used for different purposes (NHIN and member organizations)	By the Information Governance Statement of Compliance (IGSoC) and used for different purposes	By legal agreement at regional, national and international levels for the network and member organizations

**ICT**: Information and communications technology

**Table 3:** Frequency Distribution of Experts’ Opinions in the Dimension of the Components of the Suggested Framework

**Components**	**Acceptable**		**Relatively acceptable**		**Unacceptable**		**The score of each item out of 30**
	**n**	**%**	**n**	**%**	**n**	**%**	
**Leadership and coordination**	
Network leadership(Center for IT and Statistics)	7	70	3	30	0	0	27
Establishing the coordination committee from the key stakeholders	6	60	4	40	0	0	26
Establishing related committees and teams	10	100	0	0	0	0	30
**Total**	23	76.67	7	23.33	0	0	83
**Information policies**	
Compiling legal agreement for using data	9	90	1	10	0	0	29
Compiling rules for confidentiality	10	100	0	0	0	0	30
Compiling rules and policies for security	10	100	0	0	0	0	30
Compiling rules for local responsibility	10	100	0	0	0	0	30
**Total**	39	97.5	1	2.5	0	0	119
**Supplying financial and human resources**	
National and provincial supply of financial resources	6	60	3	30	1	10	25
Supplying human resources	9	90	1	10	0	0	29
**Total**	15	75	4	20	1	5	54
**Resources and infrastructure of ICT**	
Network hardware	10	100	0	0	0	0	30
Network software	8	80	2	20	0	0	28
Communicational structure of the network	6	60	4	40	0	0	26
**Total**	24	80	6	20	0	0	84
**Health indicators**							
Developing a portal for national health indicators	8	80	2	20	0	0	28
**Health data sources**	
Specifying health data sources	8	80	2	20	0	0	28
**Data management**	
Provincially via provincial networks of health information	9	90	1	10	0	0	29
Nationally via an integrated national health data repository	7	70	3	30	0	0	27
**Total**	16	80	4	20	0	0	56
**Information dissemination and use**	
Information dissemination and use at various levels	9	90	1	10	0	0	29
Using data and information for different objectives	8	80	2	20	0	0	28
Management of data exchange, share, and use	8	80	2	20	0	0	28
**Total**	25	83.33	5	16.67	0	0	85
**Total for the component dimension**	158	81.57	31	17.81	1	0.62	537

**Table 4:** Frequency Distribution of Experts’ Opinions in Dimensions of the Principles and the Architecture of the Suggested Framework

**Principles and architecture**	**Acceptable**		**Relatively acceptable**		**Unacceptable**		**The score of each item out of 30**
	**n**	**%**	**n**	**%**	**n**	**%**	
**Principles**							
Country leadership and ownership	9	90	1	10	0	0	29
Responding to country needs and demands	9	90	1	10	0	0	29
Building upon existing initiatives and systems	6	60	4	40	0	0	26
Building consensus and stakeholder involvement	8	80	2	20	0	0	28
A gradual process with a long-term vision	8	80	2	20	0	0	28
**Total for the principles dimension**	40	80	10	20	0	0	140
**Architecture**	
Members/nodes	9	90	1	10	0	0	29
Interfaces	9	90	1	10	0	0	29
Relationship with the environment	9	90	1	10	0	0	29
**Total for the architecture dimension**	27	90	3	10	0	0	87

**Table 5:** Frequency Distribution of Expert’s Opinions on the Proposed Framework

**Dimensions of the proposed framework**	**Acceptable**		**Relatively acceptable**		**Unacceptable**		**Total**	
	**n**	**%**	**n**	**%**	**n**	**%**	**n**	**%**
**The dimension of the components**	158	81.57	31	17.81	1	0.62	190	100
**The principles dimension**	40	80	10	20	0	0	50	100
**The architecture dimension**	27	90	3	10	0	0	30	100
**Total**	225	83.85	44	15.95	1	0.2	270	100
